# Transfusion in trauma: thromboelastometry-guided coagulation factor concentrate-based therapy versus standard fresh frozen plasma-based therapy

**DOI:** 10.1186/cc10078

**Published:** 2011-03-04

**Authors:** Herbert Schöchl, Ulrike Nienaber, Marc Maegele, Gerald Hochleitner, Florian Primavesi, Beatrice Steitz, Christian Arndt, Alexander Hanke, Wolfgang Voelckel, Cristina Solomon

**Affiliations:** 1 Ludwig Boltzmann Institute of Experimental and Clinical Traumatology, Donaueschingenstrasse 13, A-1200 Vienna, Austria; 2 Department of Anaesthesiology and Intensive Care, AUVA Trauma Centre, Dr. Franz-Rehrl-Platz 5, 5010 Salzburg, Austria; 3 Institute for Research in Operative Medicine, University of Witten/Herdecke, Cologne-Merheim Medical Center, Ostmerheimer Strasse 200, 51109 Cologne, Germany; 4 Department of Trauma and Orthopedic Surgery, University of Witten/Herdecke, Cologne-Merheim Medical Centre, Ostmerheimer Strasse 200, 51109 Cologne, Germany; 5 Department of Commercial Operations Western Europe, CSL Behring UK, Hayworth House, Market Place, Haywards Heath, RH16 1DB, UK; 6 Department of Anaesthesiology and Intensive Care, Salzburger Landeskliniken SALK, Müllner Hauptstrasse 48, A-5020 Salzburg, Austria; 7 Department of Anaesthesiology and Intensive Care, University Hospital Marburg, Baldingerstrasse, 35033 Marburg, Germany; 8 Department of Anaesthesiology and Intensive Care, Hannover Medical School, Carl-Neuberg Strasse 1, 30625 Hannover, Germany

## Abstract

**Introduction:**

Thromboelastometry (TEM)-guided haemostatic therapy with fibrinogen concentrate and prothrombin complex concentrate (PCC) in trauma patients may reduce the need for transfusion of red blood cells (RBC) or platelet concentrate, compared with fresh frozen plasma (FFP)-based haemostatic therapy.

**Methods:**

This retrospective analysis compared patients from the Salzburg Trauma Centre (Salzburg, Austria) treated with fibrinogen concentrate and/or PCC, but no FFP (fibrinogen-PCC group, *n *= 80), and patients from the TraumaRegister DGU receiving ≥ 2 units of FFP, but no fibrinogen concentrate/PCC (FFP group, *n *= 601). Inclusion criteria were: age 18-70 years, base deficit at admission ≥2 mmol/L, injury severity score (ISS) ≥16, abbreviated injury scale for thorax and/or abdomen and/or extremity ≥3, and for head/neck < 5.

**Results:**

For haemostatic therapy in the emergency room and during surgery, the FFP group (ISS 35.5 ± 10.5) received a median of 6 units of FFP (range: 2, 51), while the fibrinogen-PCC group (ISS 35.2 ± 12.5) received medians of 6 g of fibrinogen concentrate (range: 0, 15) and 1200 U of PCC (range: 0, 6600). RBC transfusion was avoided in 29% of patients in the fibrinogen-PCC group compared with only 3% in the FFP group (*P*< 0.001). Transfusion of platelet concentrate was avoided in 91% of patients in the fibrinogen-PCC group, compared with 56% in the FFP group (*P*< 0.001). Mortality was comparable between groups: 7.5% in the fibrinogen-PCC group and 10.0% in the FFP group (*P *= 0.69).

**Conclusions:**

TEM-guided haemostatic therapy with fibrinogen concentrate and PCC reduced the exposure of trauma patients to allogeneic blood products.

## Introduction

In patients with severe trauma, coagulopathy represents a frequent cause of death [1,2]. Prompt haemostatic intervention is necessary to prevent and correct life-threatening bleeding. Standard coagulation therapy consists of fresh frozen plasma (FFP), platelet concentrate and, in some countries, cryoprecipitate [3,4]. One approach proposed for preventing exsanguination has been to treat patients with a fixed ratio of FFP to red blood cells (RBC), but the optimal value of this ratio is still under debate [5-8]. It has been recently suggested that the time to intervention may also be an important determinant of patient outcomes [9,10].

Our group has been exploring goal-directed coagulation management using fibrinogen concentrate and prothrombin complex concentrate (PCC), administered as early as possible according to thromboelastometric (TEM) measurements [11,12]. This approach supports timely and aggressive correction of coagulopathy. It may also be considered as a strategy for reducing transfusion of allogeneic blood products: the need for FFP may be reduced by the administration of coagulation factors: fibrinogen, contained in fibrinogen concentrate, and factors II, VII, IX and X, contained in most PCCs. Furthermore, clinical and experimental data suggest that fibrinogen supplementation may also compensate for reduced platelet count [13,14]. Supplementation of fibrinogen may support primary haemostasis, because fibrinogen facilitates platelet aggregation by bridging platelet glycoprotein IIb/IIIa receptors [15]. In addition, the use of fibrinogen concentrate leads to increased firmness of the fibrin-based clot [16], whereas PCC administration may correct prolonged coagulation times through improved thrombin generation [17].

We recently reported favourable outcomes in major trauma patients referred to our level 1 trauma centre and treated following TEM-guided haemostatic therapy with fibrinogen concentrate and PCC [12]. Observed mortality in this retrospective analysis was lower than that predicted by the Revised Injury Severity Classification Score (RISC) [18] and Trauma Injury Severity Score (TRISS) [19]. The treatment strategy eliminated time delays associated with standard coagulation testing and preparation of allogeneic blood products for transfusion: more than half of the patients received haemostatic therapy within an hour of admission to the emergency room (ER). Furthermore, the low transfusion rates suggested that TEM-guided haemostatic therapy with fibrinogen concentrate and PCC may reduce the use of allogeneic blood products in trauma patients.

In the present retrospective study, we compared two different concepts of haemostatic therapy in major trauma patients: TEM-guided haemostatic therapy with fibrinogen concentrate and PCC versus FFP-based therapy. Patients receiving coagulation factor concentrates were treated at the Salzburg Trauma Centre (STC; Salzburg, Austria). Those receiving FFP-based therapy were selected from the trauma registry of the German Society for Trauma Surgery (TR-DGU), which includes 161 trauma hospitals, mostly in Germany, and holds details of a very large number of patients treated with standard coagulation therapy. We hypothesised that transfusion of RBC and platelet concentrate is lower in patients receiving TEM-guided haemostatic therapy with fibrinogen concentrate and PCC, compared with patients receiving FFP-based therapy.

We hypothesised that TEM-guided haemostatic therapy with fibrinogen concentrate and PCC may lead to increased avoidance of RBC and platelet concentrate transfusion compared with FFP-based therapy.

## Materials and methods

### Fibrinogen-PCC group (Salzburg Trauma Centre)

Following local ethics committee approval, we performed a retrospective analysis of transfusion parameters in major trauma patients who were admitted to the STC from 2006 to 2009 and treated with fibrinogen concentrate and PCC according to TEM^® ^analyses, performed using ROTEM^® ^(Tem International, Munich, Germany) as previously described by Schöchl *et al *[12]. Demographic data, laboratory data, trauma scores and outcomes data were obtained from the electronic database that was used for recording ER therapy and from the ICU database.

### FFP group (TR-DGU)

The TR-DGU is a repository for prospective, standardised and anonymous documentation of data on severely injured patients requiring ICU treatment [6]. At the beginning of 2010, TR-DGU contained data from more than 42,000 patients. Patients treated between 2005 and 2008 were included in the present study. As described elsewhere [6], the registry includes information on demographics, injury severity and pattern, pre- and in-hospital management, laboratory findings, time course and the outcome for each patient.

### Inclusion and exclusion criteria

Inclusion criteria for both groups of patients were: age between 18 and 70 years, injury severity score (ISS) of 16 or more, base deficit at admission or 2 mmol/L or higher, abbreviated injury scale (AIS) for thorax and/or abdomen and/or extremity of 3 or more and AIS for head/neck less than 5 (Table 1). Furthermore, only patients with all information needed to calculate TRISS and RISC scores were included.

**Table 1:** Inclusion criteria

	Fibrinogen-PCC group (Salzburg Trauma Centre)	FFP group (TR-DGU)
Type of therapy	ROTEM-guided administration of coagulation factor concentrates	According to local protocols
ISS	≥ 16	
AIS thorax, abdomen, extremities	At least in one region, one injury with severity degree ≥3, AIS_head/neck _< 5	
Age (years)	18-70	
Base deficit at admission	≥2 mmol/L	
FFP administered	No FFP	≥2 units FFP
Fibrinogen/PCC administered	≥1 g fibrinogen; ≥ 500 U PCC	No fibrinogen or PCC
Investigated time period	2005-2009	2005-2008
Patients included in database	353	21263
Patients fulfilling inclusion criteria	80	601

AIS, abbreviated injury scale; FFP, fresh frozen plasma; ISS, injury severity score; n, number of patients; PCC, prothrombin complex concentrate; ROTEM, thromboelastometry; TR-DGU, trauma registry of the German Society for Trauma Surgery.

For the fibrinogen-PCC group, patients who received fibrinogen (≥1 g) and/or PCC (≥500 U) but no FFP were included. For the FFP group, patients who received at least 2 units of FFP but no fibrinogen concentrate or PCC were included.

### Coagulation management

In the fibrinogen-PCC group, coagulation management was guided by TEM analysis [12]. Haemostatic therapy comprised administration of 2 to 4 g of fibrinogen concentrate (first-line therapy for patients needing increased firmness of the fibrin-based clot), and administration of 1,000 to 1,500 U of PCC, for patients showing prolonged clotting time in the thromboelastometry EXTEM test (> 1.5 times normal) [12]. This treatment was repeated as necessary. Fibrinogen concentrate was administered using two to four automatic infusion systems (Perfusor^®^, B. Braun, Melsungen, Germany) working in parallel, each at a rate of 200 mL/h; for each infusion system, 1 g of fibrinogen concentrate was diluted in 50 mL of water for injections. The resulting administration rate was 2 to 4 g in 15 minutes. For patients in whom fibrinogen could not fully compensate for decreased platelet levels, platelet concentrate was transfused (platelet concentrate was recommended if the EXTEM-MCF is decreased to < 40 mm when FIBTEM-MCF is 10 to 12 mm). The target haemoglobin concentration during the operative procedure was 10 g/dL. In the postoperative phase, lower haemoglobin levels were tolerated.

Coagulation management of patients in the FFP group was dictated by clinical practice at each trauma department and was therefore not standardised. TEM is not used in standard practice; nevertheless, isolated use in some hospitals means that a minority of patients in the registry may have been treated with some TEM guidance. Although the treatment of patients in the TR-DGU is not standardised, it represents the general approach to coagulation management of major trauma patients in Germany, with FFP administered as first-line haemostatic therapy, and platelet concentrate and RBC used as necessary. Laboratory analyses of coagulation were performed in the local laboratories; the register collects no information on the type of analyses, reagents or devices on which they are performed, or on their role in guiding haemostatic therapy within the local protocol.

### Selection of variables for analysis

For all subjects, age and gender were documented together with the following parameters upon admission: coagulation results, blood pressure, heart rate, temperature, ISS and Glasgow coma scale score. Predicted mortality for each patient was estimated using the RISC and the TRISS methodology. Mortality rate (until discharge from the hospital) was documented.

Details of coagulation management were noted for the acute phase (ER and early surgery phase) and the first 48 hours spent in the ICU. For the fibrinogen-PCC group, administration of RBC, fibrinogen concentrate, PCC and platelet concentrate were noted for both time periods. For the FFP group, administration of RBC and FFP were noted for both time periods; data for platelet concentrate administration were only available for the acute phase.

### Statistical analysis

Demographic and clinical data were presented as mean ± standard deviation or median (minimum, maximum or interquartile range (IQR), as indicated) for continuous variables, and as percentages for categorical variables. For continuous variables, normal distribution was analysed by the Shapiro-Wilk test. To detect differences between the patient groups, either the Student's *t*-test or the Mann-Whitney *U* test was performed, depending on the underlying distribution. For categorical variables, Fisher's exact test was used. Statistics were calculated using IBM SPSS Statistics 18 (SPSS Inc., Chicago, IL, USA).

## Results

In the fibrinogen-PCC group, 80 of 353 patients treated in the STC between 2005 and 2009 fulfilled the inclusion criteria. Between 2005 and 2008 (data for 2009 were not available at the time of analysis), 21,263 patients were included in the TR-DGU. Of 21,263 patients, 2,582 fulfilled the general inclusion criteria. At this step, most cases were lost due to missing base deficit values. After applying the specific haemostatic therapy criteria (Table 1), 601 patients could be included in the FFP group.

Demographic data and trauma scores were available for all patients included in the study. As intended, the two groups were comparable with regard to demographic parameters as well as the overall magnitude of injury sustained and probability of survival assessed by the TRISS and RISC scores (Table 2). With regard to the pattern of injury, patients in the FFP group had sustained head and thoracic injuries of higher magnitude, whereas fibrinogen-PCC patients had sustained more severe abdominal injuries. Patients in the fibrinogen-PCC group also appeared to be less haemodynamically stable upon arrival at the ER. Standard laboratory coagulation data were available for at least 90% of the patients included in the study. A significantly more prolonged prothrombin time (PT) was observed in the fibrinogen-PCC group (*P *= 0.0001; Table 3); this difference was apparent upon arrival at the ICU as well as the ER. The base deficit also differed between the groups (6.4 ± 3.4 in the fibrinogen-PCC group and 6.9 ± 4.5 mmol/L in the FFP group), but this difference did not reach statistical significance.

**Table 2:** Patient demographic and clinical data

	Fibrinogen-PCC group (*n *= 80)	FFP group (*n *= 601)
Age (years)	37.3 ± 14.5	39.1 ± 14.5
Male *n* (%)	63 (79)	442 (74)
Systolic blood pressure on admission at ER (mmHg)	95 ± 30	108 ± 30**
Heart rate on admission at ER (beats/minute)	105 ± 26	99 ± 24*
ISS	35.5 ± 10.5	35.2 ± 12.5
GCS	12.2 ± 3.4	11.3 ± 4.4*
AIS Head	1.1 ± 1.5	1.6 ± 1.7*
AIS Chest	2.1 ± 2.0	3.1 ± 1.7**
AIS Abdomen	2.5 ± 2.1	2.1 ± 1.8
AIS Extremity	2.9 ± 1.8	2.9 ± 1.4
TASH	13.9 ± 6	12.6 ± 5*
RISC	6.9 (2.4, 16.2)	8.5 (3.3, 24.8)
TRISS	13 (3, 38)	7 (2, 38)
Death	6 (7.5)	60 (10)

Data are presented as mean ± standard deviation, median (interquartile range) or as absolute and relative frequency.

*P< 0.05, significantly different than the fibrinogen-PCC group;

**P = 0.0001, significantly different than the fibrinogen-PCC group.

AIS, abbreviated injury score; ER, emergency room; FFP, fresh frozen plasma; n, number of patients; GCS, Glasgow coma scale; ISS, injury severity score; PCC, prothrombin complex concentrate; RISC, revised injury severity classification; TASH, trauma associated severe haemorrhage score; TRISS, trauma injury severity score.

**Table 3:** Standard laboratory parameters

	Admission in the emergency room		Arrival at the ICU	
		
	Fibrinogen-PCC group (*n *= 80)	FFP group (*n *= 601)	Fibrinogen-PCC group (*n *= 80)	FFP group (*n *= 601)
Haemoglobin (13.5-17 g/dL)	9.9 ± 3.1	9.6 ± 2.6	9.6 ± 2.1	9.7 ± 1.9
Platelet count (150-350 *1000 cells/μL)	178 ± 68	184 ± 79	105 ± 58	108 ± 60
Prothrombin time in percentage (70-110%)	59 ± 19	65 ± 21*	52 ± 16	71 ± 18**
Fibrinogen (2-4.5 g/L)	1.4 ± 0.7	not available	1.8 ± 0.5	not available

Data are presented as mean ± standard deviation; normal range is indicated in parentheses. Data available for at least 90% of patients.

*P< 0.05, significantly different than the fibrinogen-PCC group;

**P = 0.0001, significantly different than the fibrinogen-PCC group. FFP, fresh frozen plasma; *n*, number of patients; PCC, prothrombin complex concentrate.

**Figure 1:**
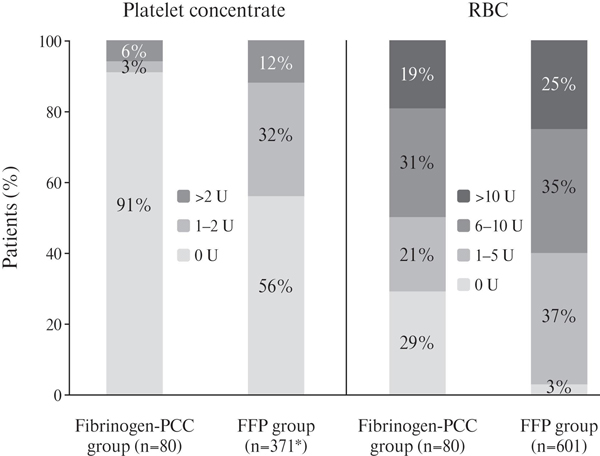
**Platelet concentrate and red blood cell (RBC) transfusion in the emergency room and during surgery**. *Platelet concentrate transfusion only reported for 371 of 601 patients from the trauma registry of the German Society for Trauma Surgery (TR-DGU). FFP, fresh frozen plasma; PCC, prothrombin complex concentrate.

RBC transfusion data for treatment in the ER and during surgery were available for all patients. Complete avoidance of RBC transfusion was observed in 3% of patients in the FFP group and 29% of patients in the fibrinogen-PCC group (*P*< 0.0001; Figure 1). In the FFP group, 583 of 601 patients (97%) received RBC transfusion (number of units ranging between 1 and 64), compared with 57 of 80 patients (71%) in the fibrinogen-PCC group (range: 1 to 28 units). Information on platelet concentrate transfusion for treatment in the ER and during surgery was available for 371 of the 601 patients in the FFP group, and for all patients in the fibrinogen-PCC group. Platelet concentrate was administered to 44% of patients in the FFP group, compared with 9% in the fibrinogen-PCC group (*P *= 0.0001; Figure 1). Of interest, all patients with no transfusion of RBC also did not receive any platelet concentrate, meaning total avoidance of allogeneic blood products in 29% of the patients in the fibrinogen-PCC group.

**Figure 2:**
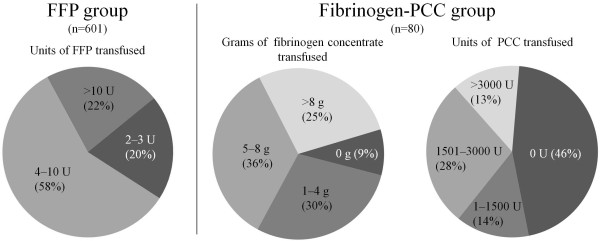
**Percentage of patients receiving the indicated amount of haemostatic agent (FFP, fibrinogen concentrate, PCC) in the emergency room and during surgery**. Percentage of patients in brackets. FFP, fresh frozen plasma; PCC, prothrombin complex concentrate.

For haemostatic therapy in the ER and during surgery, the FFP group received a median of 6 units of FFP (IQR: 4, 10; range: 2, 51), 6 units of RBC (IQR: 4, 11) and 0 units of platelet concentrate (IQR: 0, 2; range: 0, 8). The patients in the fibrinogen-PCC group received a median of 6 g of fibrinogen concentrate (IQR: 3, 9; range: 0, 15) and 1,200 IU of PCC (IQR: 0, 2,400; range: 0, 6,600), while RBC median transfusion was 5.5 units (IQR: 0, 9.5) and platelet concentrate median transfusion was 0 units (IQR: 0, 0; range: 0, 2). The dosage of FFP, fibrinogen and PCC is described in Figure 2. During the first 48 hours after admission to the ICU, patients in the FFP group received median doses of 3 units of RBC (IQR: 1, 6; range: 0, 80; data reported for 424 patients) and 3 units of FFP (IQR: 0, 6; range: 0, 80; data reported for 405 patients). No information is available on platelet concentrate transfusion in this group during the stay on the ICU. For the fibrinogen-PCC group, a complete set of transfusion data was available. During their stay at the ICU, these patients received a median dose of 2 units of RBC (IQR: 0, 3; range: 0, 11), platelet concentrate was transfused in 9% of the patients during this time (the dose was 1 or 2 units). The patients also received a median dose of 6 g of fibrinogen concentrate (IQR: 3, 10; range: 0, 22) and a median of 1,200 U of PCC (IQR: 0, 2,400; range: 0, 9,000).

**Figure 3:**
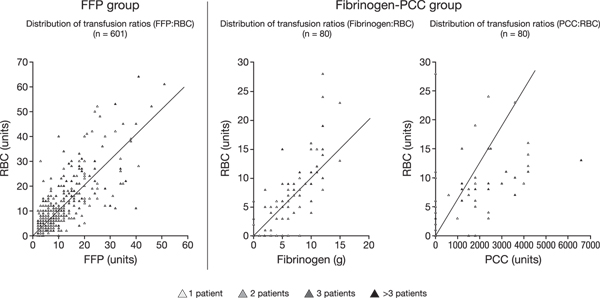
**Distribution of transfusion ratios in the FFP and fibrinogen-PCC groups (data are for treatment in the emergency room and during surgery)**. The line shown on each graph represents the median ratio (FFP:RBC 1; fibrinogen:RBC 0.9; PCC [in hundreds of units]:RBC 1.6). FFP, fresh frozen plasma; PCC, prothrombin complex concentrate.

For haemostatic therapy in the ER and during surgery, the median ratio of FFP:RBC in the FFP group was 1 (IQR: 0.7, 1.3; minimum 0.1, maximum 6.5); Figure 3 shows the ratios for all patients in the group. The same median value was observed in each of the four years included in the analysis (2005 to 2008). In the fibrinogen-PCC group, the median ratio of fibrinogen concentrate:RBC for coagulation therapy in the ER and during surgery was 0.9 (IQR: 0.7, 1.2), and the ratio of PCC (in hundreds of units):RBC was 1.6 (IQR: 0, 3); Figure 3 shows the distributions of fibrinogen concentrate:RBC and PCC:RBC ratios.

Data relating to subsequent outcomes were available for all patients. The median duration of postoperative intubation was 9.5 days (IQR: 3.5, 14) in the fibrinogen-PCC group, significantly longer than the 7 days (IQR: 2, 16) in the FFP group (*P *= 0.005). Median length of stay (LOS) in the ICU, however, was comparable in the two groups: 14.5 days (IQR: 8.5, 21) in the fibrinogen-PCC group and 14 days (IQR: 6, 23) in the FFP group (*P *= 0.95). In contrast, the median LOS in the hospital was significantly different between the two groups: 23 days (IQR: 14.5, 40.5) in the fibrinogen-PCC group and 32 days (IQR: 20, 49) in the FFP group (*P *= 0.005). Mortality was 10.0% in the FFP group and 7.5% in the fibrinogen-PCC group (*P *= 0.69, not significant).

## Discussion

The present study compared TEM-guided haemostatic therapy using fibrinogen concentrate and PCC, with standard FFP-based therapy, in trauma patients. RBC transfusion was avoided in 29% of patients in the fibrinogen-PCC group, and these patients received no transfusion of any allogeneic blood products. In contrast, RBC transfusion was avoided in only 3% of patients in the FFP group. Transfusion of platelet concentrate was avoided in 91% of patients in the fibrinogen-PCC group, compared with 56% in the FFP group. In our trauma centre, TEM-guided haemostatic therapy with fibrinogen concentrate and PCC has been associated with a continuing decrease in the use of all types of allogeneic blood products.

Minimising or avoiding exposure to allogeneic blood products is clearly desirable. The reasons for developing alternative treatments include intermittent supply shortages and public concern regarding the safety of allogeneic blood products [20,21]. Transfusion of FFP, for instance, carries the risk of transfusion-related lung injury, transfusion-associated circulatory overload, acute respiratory distress syndrome, transfusion-related immunomodulation and pathogen transmission [22-24]. Attempts to reduce FFP transfusion are complicated by the fact that small quantities of FFP are not effective in correcting coagulopathy [25,26]. Therefore, administration of FFP in larger amounts should be recommended, but high doses may have a dilutional effect on haematocrit, leading to an increase in RBC transfusion. In contrast, our study showed a reduction in RBC and platelet concentrate transfusion among patients treated with fibrinogen concentrate and PCCs. High levels of fibrinogen increase maximum clot firmness even in patients with a low platelet count, suggesting possible compensation for reduced platelet levels (it is hypothesised that an increased number of fibrin molecules binding a smaller number of platelets may be feasible without compromising clot integrity) [13,14]. The explanation for reduced RBC transfusion is more uncertain, but coagulation factor concentrates may provide faster cessation of bleeding and reduced haemodilution compared with allogeneic blood products. Due to their low volume of administration, coagulation factor concentrates are also likely to have a smaller effect on haematocrit. The use of TEM to diagnose coagulopathy may additionally help reduce RBC and platelet concentrate transfusion. There is increasing evidence of the usefulness of viscoelastic methods for diagnosing trauma-induced coagulopathy [12,27-29], and several reports have described a reduction in transfusion requirements following its introduction to treatment algorithms [30-32].

Our approach to managing coagulopathy in trauma patients focuses on the use of fibrinogen concentrate and PCC, which are quicker to administer than allogeneic blood products. Several groups have suggested that reducing the time to administer haemostatic therapy may improve patient outcomes [8-10]. Our group recently described an algorithm of goal-directed coagulation therapy with fibrinogen and PCC in major trauma patients [12], and in that study 52% of patients received the first dose of fibrinogen concentrate within the first hour, most of them within 30 minutes. In contrast, in a study published by Snyder *et al*., the first unit of FFP was typically administered at a median of 93 minutes after arrival at the ER [8]. Such delay may be related to the need for blood group matching, thawing and warming of FFP before administration (thawing and warming usually take about 30 minutes). It may be possible to address this delay, for example by storing thawed plasma for immediate application [33]. The use of pre-defined transfusion packages has also been described [34]. Most trauma centres use defined transfusion packages containing cooled RBC and frozen or thawed FFP. Unfortunately, thawing FFP in advance may have negative consequences, because unused thawed units must be discarded. To reduce apparent wastage, physicians may be tempted to overuse FFP. This tendency must be considered in the context of today's economic and administrative pressures, because the costs of blood products are high and often underestimated [35]. The time to infuse medication is another consideration. In general, it is recommended that one unit of FFP is administered over a period of about 30 minutes. In contrast, typical doses of fibrinogen concentrate and PCC may be administered in less than 10 minutes [16,36], and plasma levels of the coagulation factors administered rise rapidly after infusion.

This study was not designed to establish whether TEM-guided haemostatic therapy with fibrinogen concentrate and PCC improves mortality. Large numbers of patients would be required to provide statistically robust evidence on mortality [37]. We nevertheless report an encouraging trend towards lower mortality in the fibrinogen-PCC group compared with the FFP group: 7.5% versus 10.0% (*P *= 0.69). One factor likely to affect survival is the speed of administration of haemostatic therapy - as discussed above, TEM-guided haemostatic therapy with fibrinogen concentrate and PCC may be advantageous from that point of view. The quantity of fibrinogen administered may also affect mortality rates. Stinger *et al*. reported correlations between the amount of fibrinogen administered and blood loss and survival in severely bleeding patients from the Iraq war [38]. Successful haemostatic therapy with fibrinogen concentrate has been described in other settings involving extensive surgery and blood loss (e.g., cardiovascular surgery) [39-41]. Successful use of PCC to treat acquired coagulopathy in the perioperative setting has previously been reported, albeit in limited numbers of patients [11,12,42,43]. Animal experiments have suggested that PCC may be more effective than FFP in the trauma setting [44], whereas Austrian guidelines recommend PCC administration in bleeding patients if clotting time measured by thrombelastography (TEG)/TEM is prolonged [45]. In the present study, PCC was administered to treat bleeding when clotting time in the EXTEM assay was prolonged.

The study inclusion criteria aimed at minimise between-group differences in patient characteristics. The choice of 1 g fibrinogen/500 U PCC as inclusion criteria was based on practical therapy. The minimum amount of fibrinogen concentrate administered in clinical practice is 1 g, and patients from the STC were eligible for inclusion in the study once they had received this dose. Similarly, the minimum dose of PCC was 500 U. We chose 2 units of FFP as the criterion for the comparator group because this dose should contain approximately 1 g of fibrinogen [4], thus enabling comparison with the fibrinogen-PCC group.

The data analysis revealed some between-group differences in patient characteristics, and these are worthy of consideration. Although ISS, TRISS, RISC and AIS for abdomen and extremity were not significantly different, there was a significant trend towards more severe head and chest trauma in the FFP-group. Surprisingly, however, the score predicting massive transfusion (TASH) was higher in the fibrinogen-PCC group. Furthermore, it is difficult to estimate whether trauma-induced coagulopathy related to hypoperfusion was more pronounced in either of these two groups. On the one hand, blood pressure was significantly lower in the fibrinogen-PCC group, and base deficit was non-significantly lower in this group. On the other hand, both PT (expressed as a percentage) and platelet count were higher in the FFP group (*P *not significant for platelet count). Had hypoperfusion been more pronounced in the fibrinogen-PCC group, the significantly lower transfusion rates would appear even more encouraging.

The present study has several limitations. Data for the fibrinogen-PCC group were collected retrospectively from only one centre. TR-DGU data are collected via standardised forms from trauma centres throughout central Europe. Although only the main parameters of trauma management and patient outcome are reported and the collection of data was carefully checked, there may be some reporting bias. Furthermore, for some patients included in the study, the data were incomplete - particularly regarding platelet concentrate transfusion. It cannot be ruled out that some centres providing data to the TR-DGU may be using TEM sporadically. As there are currently no publications on the use of TEM in these centres, the impact on our results is difficult to estimate. The present study did not evaluate any safety aspects, such as thromboembolic or infectious complications. The important difference observed in LOS in the hospital between the two groups, although encouraging, may be influenced by local patient management protocols. A prospective study would be needed to confirm which therapeutic approach offers the more favourable outcome.

## Conclusions

In the present study, TEM-guided haemostatic therapy with fibrinogen concentrate and PCC reduced the exposure of trauma patients to allogeneic blood products. To improve current transfusion practice, the potential role of coagulation factor concentrates in achieving haemostasis rapidly among trauma patients must be considered.

## Key messages

• In attempting to reduce transfusion of allogeneic blood products, new therapeutic options are being investigated for the management of bleeding in trauma patients.

• The present study compared transfusion of RBC and platelet concentrate in patients receiving either TEM-guided haemostatic therapy with fibrinogen concentrate and PCC, or standard FFP-based therapy.

• RBC transfusion was avoided in 29% of patients in the fibrinogen-PCC group, and these patients received no transfusion of any allogeneic blood products. In contrast, RBC transfusion was avoided in only 3% of patients in the FFP group.

• Transfusion of platelet concentrate was avoided in 91% of patients in the fibrinogen-PCC group, compared with 56% in the FFP group.

• TEM-guided haemostatic therapy with fibrinogen concentrate and PCC reduced the exposure of trauma patients to allogeneic blood products.

## Abbreviations

AIS: abbreviated injury score; ER: emergency room; FFP: fresh frozen plasma; IQR: interquartile range; ISS: injury severity scores; LOS: length of stay; PCC: prothrombin complex concentrate; PT: prothrombin time; RBC: red blood cells; RISC: revised injury severity classification score; STC: Salzburg Trauma Centre; TEM: thromboelastometry; TR-DGU: TraumaRegister DGU; TRISS: trauma injury severity score.

## Competing interests

This study was performed without external funding. HS, CS and MM have received honoraria as speakers and research support from CSL Behring (manufacturer of fibrinogen concentrate and PCC) and Tem International GmbH (manufacturer of the TEM device). AH has received honoraria as speaker and research support from CSL Behring. GH is an employee of CSL Behring. All other authors declare that they have no competing interests.

## Authors' contributions

HS designed the study, contributed to acquiring the data in the STC, and wrote the manuscript. UN acquired the data from the TR-DGU, performed the statistical analysis and contributed to writing the manuscript. MM contributed to acquiring the data from the TR-DGU and to writing the manuscript. GH contributed to designing the study, analysing the data and writing the manuscript. FP, BS and CA acquired the data from the STC. AH, WV and CJ contributed to writing the manuscript. CS contributed to designing the study, analysing the data, and writing the manuscript. All authors read and approved the final manuscript.
